# Vesico-Vaginal Fistula

**Published:** 1867-10

**Authors:** C. C. F. Gay

**Affiliations:** One of the Surgeons of the Buffalo General Hospital


ART. II.—*Vesico-Vaginal Fistula.* BY C. C. F. GAY, M. D., *one of the Surgeons of the Buffalo General Hospital.*

The surgical treatment of the unfortunate female who is, or may be the victim of the terrible result of her accouchment styled vesico-vaginal fistula, has been brought to such degree of perfection in our own day, by means of appliances and instruments devised by our distinguished countryman, Dr. Sims, with the aid and assistance of his no less worthy successor, at present surgeon of the Woman's Hospital, New York, Dr. Thomas Addis Emmet, that almost any surgeon possessing the requisite tact in the use of instruments need ever despair of obtaining satisfactory results in any case to which he may be called upon to operate.

It is truly wonderful what measure of progress has been made during the last decade for the relief and permanent cure of this unfortunate condition of the female, but so recently supposed to be a condition irremediable. Credit must be awarded in the first instance to those benevolent women of New York, who first moved in the enterprise which resulted in the establishment of an hospital to be devoted exclusively to the treatment of diseases of their own sex.

This act upon the part of the women of New York provided a field hitherto almost unexplored, wherein the rare gifts and genius of Sims could have free scope. In the absence of these initiatory steps by the charitable of New York, Sims would have been unable to contribute so largely of his experience to the profession of this and the old country. The opportunity was thus created and given to him, and to show how well he has improved the opportunity, reference only need be made to his words and works. We would say naught to derogate from the great merits of Sims, nor be unmindful of the merit of those who by their kindness and liberality opened up the way whereby the former could exercise his rare gifts and promulgate his discoveries and improvements to the world. All honor to the noble women and liberal men of New York, and all honor to Sims!

This paper proposes only to give some general observations upon the operation for vesico-vaginal fistula, and in a future paper we design to record somewhat in detail some of the difficulties attending the various steps of the operation, to show that some of those difficulties are only apparent, and not real, and to describe how those little difficulties have been overcome by us during the progress of an operation without any fore-knowledge or fore-warning as to the difficulties besetting the way when undertaking the operation.

At the close of an operation recently made in the country, our assistant was led to remark that "after all an egg was easily made to stand upon its end." The greatest difficulty I apprehend in the operation next to that of the necessary preparation of the parts for the operation, is the proper denudation of the fistulous border. Some patients require weeks of preliminary preparation, while others need none at all; if the cul-de-sac be preserved preliminary preparation need not occupy much time; if destroyed then initiatory or operations are frequent. Position is all-important. If practicable the best position is that upon the knees, with the head low. We first saw the advantage of this position while assisting our friend, Dr. Ring, in an operation upon a very obese subject. In this position the fore-finger of the left hand could easily be introduced into the fistula, upon which its uterine border could be raised, held firmly, and the mucous membrane denuded with ease, and with more facility than when raised by the tenaculum. The advantage of this procedure, of holding the parts to be denuded over the usual method by the tenaculum, was first made apparent and practicable by ourself when denuding the mucous membrane in the operation for complete laceration of perenium, the distance from the vaginal outlet being about the same in both cases; this method of procedure is not of course practicable in every case, on account of the distance of the fistula from the outlet, but when not too remote the parts may be manipulated with great facility, and when the scissors are used the margin may be pared very quickly.

Assisting in an operation some three or four years since, I recommended and urged the use of the interrupted suture, but instead thereof the clamp was used, the operation being success-ful. But at that time I could not see, neither can I now, any advantage of the clamp over the interrupted suture. The clamp complicates the operation and prolongs it greatly, and time, I conceive, when a woman is in the position named above, is to be taken into account. The parts properly prepared, and the denuded surfaces brought in apposition, the interrupted silver suture will as certainly retain the parts in apposition as well the clamp, provided, always, a sufficient number are used.

In assisting those who have given preference to the long needle, I have been struck forcibly with its great inconvenience, the short needle armed with the silk loop, and used with forceps, is much to be preferred. I have seen one case where the Sims' catheter, or no other catheter could be retained in the bladder on account of the great irritation produced by them when in situ. It was necessary to introduce the gum elastic catheter every few hours, day and night. The operator can readily see, therefore, how important it is that all source of irritation from this quarter be allayed previous to operating.

In all uterine surgical operations, and indeed in all operations of the female genital organs, Dr. Emmet of New York is emphatic in stating his preference for the use of the scissors over that of the knife. I am not sure but the preference for one or the other of these instruments might better be left with the operator than to insist upon the use of the scissors as the superior cutting instrument to the knife, or its *par excellence.*

I am not certain but one surgeon may be more dextrous in the use of the scissors and another surgeon more dextrous in the use of the knife. For my own part I have no difficulty in deciding which to use. In my own hands the scissor is incomparably superior to the knife, and I must say here that I used them in operations involving the female genital organs before it became known to me that they were used or preferred by any other surgeon. I would like to say more upon the subject of the scissors, their shape, etc., but must reserve the further consideration of this topic for a future paper, and as I have already occupied as much space as I should in speaking of the operation of vesico-vaginal fistula, in a general way, will reserve what I have further to say of the operation in detail, giving the result of two cases, in one of which the clamp and in the other the interrupted suture was used, and both equally successful after the first operation.

